# Worldwide population distribution of the common LCE3C-LCE3B deletion associated with psoriasis and other autoimmune disorders

**DOI:** 10.1186/1471-2164-14-261

**Published:** 2013-04-17

**Authors:** Laia Bassaganyas, Eva Riveira-Muñoz, Manel García-Aragonés, Juan R González, Mario Cáceres, Lluís Armengol, Xavier Estivill

**Affiliations:** 1 Genetic Causes of Disease Group, Centre for Genomic Regulation (CRG), Barcelona, E-08003, Spain; 2 Universitat Pompeu Fabra (UPF), Barcelona, E-08003, Spain; 3 Hospital del Mar Medical Research Institute (IMIM), Barcelona, E-08003, Spain; 4 Centro de Investigación Biomédica en Red en Epidemiología y Salud Pública (CIBERESP), Barcelona, E-08003, Spain; 5 Centre de Recerca en Epidemiologia Ambiental (CREAL), Barcelona, E-08003, Spain; 6 Laboratori de Retrovirología, IrsiCaixa, Hospital Germans Trias i Pujol, Barcelona, Badalona, 08916, Spain; 7 qGenomics Laboratories, Barcelona, E-08003, Spain; 8 Institut de Biotecnologia i de Biomedicina, Universitat Autònoma de Barcelona, Bellaterra, E-08193, Spain; 9 Institució Catalana de Recerca i Estudis Avançats (ICREA), Barcelona, E-08010, Spain

**Keywords:** Copy number variants, Psoriasis, Autoimmune disorders, *LCE3C_LCE3B-del*, Genetic variation, Genetic drift, Human populations

## Abstract

**Background:**

There is increasing evidence of the importance of copy number variants (CNV) in genetic diversity among individuals and populations, as well as in some common genetic diseases. We previously characterized a common 32-kb insertion/deletion variant of the *PSORS4* locus at chromosome 1q21 that harbours the *LCE3C* and *LCE3B* genes. This variant allele *(LCE3C_LCE3B-del)* is common in patients with psoriasis and other autoimmune disorders from certain ethnic groups.

**Results:**

Using array-CGH (Agilent 244 K) in samples from the HapMap and Human Genome Diversity Panel (HGDP) collections, we identified 54 regions showing population differences in comparison to Africans. We provided here a comprehensive population-genetic analysis of one of these regions, which involves the 32-kb deletion of the *PSORS4* locus. By a PCR-based genotyping assay we characterised the profiles of the *LCE3C_LCE3B-del* and the linkage disequilibrium (LD) pattern between the variant allele and the tag SNP rs4112788. Our results show that most populations tend to have a higher frequency of the deleted allele than Sub-Saharan Africans. Furthermore, we found strong LD between rs4112788*G* and *LCE3C_LCE3B-del* in most non-African populations (r2 >0.8), in contrast to the low concordance between loci (r2 <0.3) in the African populations.

**Conclusions:**

These results are another example of population variability in terms of biomedical interesting CNV. The frequency distribution of the *LCE3C_LCE3B-del* allele and the LD pattern across populations suggest that the differences between ethnic groups might not be due to natural selection, but the consequence of genetic drift caused by the strong bottleneck that occurred during “out of Africa” expansion.

## Background

Structural variants, largely represented by copy number variants (CNV), are a rich source of genetic polymorphism and they may potentially have a strong impact on genetic diversity among individuals [1-3]. The biological importance of CNV has become increasingly apparent through the application of various comprehensive and complementary approaches to analyse CNV, including array-comparative genomic hybridization (aCGH) and more recently, next-generation sequencing (NGS) technologies [2-9]. The functional impact of CNV has been demonstrated at all biological levels [10], from cellular effects on gene expression [11] to their association with several types of complex traits and genetic diseases [12-17], as well as with different types of cancers [18-20].

Human population genetics studies allow major population branches and subpopulation groups to be defined [21], for which excellent resources are now available, such the Human Genome Diversity Panel (HGDP) [22] and HapMap Collection [23]. The analyses of the human genome at the nucleotide and structural levels have shown that genetic clusters closely correspond to groups defined by ethnicity or continental ancestry [24]. Moreover, several studies have reported CNV containing regions that show population differences in copy number [25-27]. These examples suggest that genetic differences between ethnic groups involving large genomic regions affect functional elements influenced by the environment, which are therefore potential substrates for natural selection.

We recently characterized a common 32-kb deletion in the *PSORS4* locus on chromosome 1q21 that harbours the *LCE3C* and *LCE3B* genes. The deleted allele (*LCE3C*_*LCE3B-del)* is common in patients with psoriasis among populations of European ancestry [16,28,29], and in Chinese and Mongolian populations [29-31]. In addition, successive studies also found this deletion to be associated to rheumatoid arthritis in Spanish and Chinese patients [32,33], psoriatic arthritis in Spanish and Italian populations [34], and systemic lupus erythematous in Chinese patients [33].

Differences in the frequency of *LCE3C*_*LCE3B-del* and its relationship to disease in different ethnic groups suggest a possible role of environmental factors and demographic history on this polymorphic deletion and on its associated diseases. In fact, the diseases associated with *LCE3C*_*LCE3B-del* are generally regarded as immunological disorders, the aetiology of which is influenced by both environmental (infection, drugs, stress and climate) and genetic factors [35]. As such, we might expect a variable frequency of the polymorphisms around the world. By better understanding the genetic variability of *LCE3C*_*LCE3B-del* in populations associated with distinct environments and with other specific demographic histories, we might gain insight into the interaction between some of the multiple components involved in several complex diseases.

Here we have used array comparative genomic hybridisation (aCGH) to characterize the profile of inter-population differences of the *LCE3C_LCE3B-del* allele in 13 population groups from the HapMap and Human Genome Diversity Panel (HGDP) collections, and of the *LCE3C_LCE3B-del* and tag SNP rs4112788 association [16] across 31 ethnic groups from the HGDP. The results provide a comprehensive view of the population distribution of this common functional CNV, suggesting that genetic drift caused by strong bottleneck occurred during the “out of Africa” has established this common deletion at high frequencies in most of non-Sub-Saharan African populations.

## Results

### Identification of population specific CNV using aCGH

Following the criteria described for CNV detection (see Additional file 1), we observed 54 regions at least 30-kb long whose signal intensities (i.e. copy number) indicated a distinct distribution in at least one of the 12 populations when compared to the Yoruba (YRI) population (Additional file 2: Figure S1 and Additional file 3: Table S3). Populations from Eastern-Asia, America and Oceania had greater differences in signal intensity with respect to the YRI population than other Sub-Saharan African, European, Northern African and Middle East populations, as well as more copy-number variable regions (Additional file 2: Figure S2), consistent with previous studies of human populations [24,36-40].

All 54 regions for which a difference in intensity could be observed in a population corresponded to known CNV described in the Database of Genomic Variants (http://projects.tcag.ca/variation/). Of these 54 regions, 36 (67%) totally or partially overlapped with 58 RefSeq genes, indicating that the variation in CNV between population groups involves functional elements, in agreement with previous data, and indicating a significant relationship between the genomic regions affected by CNV and gene content [1,3]. In addition, 39 out of 54 regions (72%) are enriched in segmental duplications (SD), also consistent with previous studies [1,3,5,41,42] (Additional file 3: Table S3).

We first carried out a Gene Ontology (GO) analysis (http://www.geneontology.org/) of the variable genomic regions that contain known genes to select categories of enriched genes with p-values below 0.01. Our set of genes was significantly enriched in proteins related to the response to environmental stimulus (sensory perception of chemical stimulus [adjP = 0.0002]), the immune system (antigen processing and presentation [adjP = 0.0002]) and metabolism (carboxylase activity [adjP = 0.007]). Relaxing the p-values to 0.05, we also found enrichment in keratinisation (adjP = 0.0352), the category represented by *LCE3C* and *LCE3B* genes.

Several CNV that show variable signal intensity across populations contain genes known to be associated with disease (Additional file 3: Table S3), indicating that population adaptation to different environments could entail effects on pathological mechanisms. Although in this study we focused on the variability of the *LCE3C*_*LCE3B-del* among populations due to its association with the susceptibility to psoriasis and other autoimmune diseases in some ethnic groups, other copy number variant regions contain genes that deserve further attention at the population genetics level. For example, *CFHR3* and *CFHR1* are associated with age-related macular degeneration [43-45] and systemic lupus erythematous [46]. *GSTT1* has been linked to breast cancer [47], *NAFLD* to non-alcoholic fatty liver disease [48], and *HLA-DRB5* and *HLA-DQA1* to autoimmune diseases [49,50]. Due to their functional characteristics, most of these genes are very appealing from both biomedical and evolutionary perspectives, supporting the idea that the variable prevalence of some diseases among ethnic groups is in part linked to environmental conditions.

### Analysis of the *LCE3C_LCE3B-del* frequency in worldwide populations

The aCGH data for the *LCE3C_LCE3B-del* showed lower intensity values for all the studied populations respect to YRI, and five of them (Pima (PIMA), Brahui (BRA), Mozabite (ALG), Maya (MAYA) and Chinese Han (CHB)) presented log_2_ ratios ≤0.25, limit considered significantly low for a copy-number loss by our algorithm (Table 1, Additional file 1). This suggests that not only there is a high frequency of the deletion in European and Asian populations when compared to Africans, as shown previously [16,30,31], but other population groups are also likely to have higher frequencies of the *LCE3C_LCE3B-del.*

**Table 1:** **Comparison of *LCE3C_LCE3B-del *aCGH values with *LCE3C_LCE3B-del *and rs4112788 genotypes in 13 world populations**

			**LCE3C/LCE3B genotype frequency (%)**			**LCE3C/LCE3B allele frequency (%)**		**rs4112788 genotype frequency (%)**		
**Geographic Region**	**Population (ID)**	aCGH intensity values (respect to YRI*)	**+/+**	**+/−**	**−/−**	**+**	**−**	**AA**	**AG**	**GG**
Sub-Saharan Africans	Pygmies* (PYG)	−0.13	38.6	52.3	9.1	64.77	35.23	0	13.64	86.36
	Yoruba (YRI)	0*	36.4	59.1	4.5	65.91	34.09	0	28.57	71.43
	Bantu (BAN)	−0.03	44.4	55.6	-	72.22	27.78	0	50.00	50.00
North-Africans/Middle East	Mozabite*^#^ (ALG)	−0.3	11.5	42.3	46.2	32.69	67.31	0	21.43	78.57
	Bedouin* (BED)	−0.21	19.4	55.6	25	47.22	52.78	14.63	53.66	31.71
Europians	French (FRA)	−0.2	22.2	37	40.7	40.74	59.26	16.67	41.67	41.67
Southern Asians	Brahui^#^ (BRA)	−0.31	22.7	40.9	36.4	43.18	56.82	21.74	43.48	34.78
	Hazara (HAZ)	−0.15	58.9	41.1	0	79.41	20.59	43.75	56.25	0
Eastern Asians	Han^#^ (CHB)	−0.26	14.7	47.1	38.2	38.24	61.76	0	52.17	47.83
	Yakut* (YAK)	−0.12	63.6	27.3	9.1	77.27	22.73	50.00	31.82	18.18
Oceania	Papuan-Melanesian (OCE)	−0.18	31.4	37.1	31.4	50.00	50.00	26.47	41.18	32.35
Americans	PIMA^#^	−0.67	9.1	31.9	59.1	25.00	75.00	9.09	36.36	54.55
	MAYA^#^	−0.27	9.5	57.1	33.3	38.10	61.90	7.14	57.14	35.71

Comparison of aCGH log_2_ ratio values for each population analysed in the array against the Yoruba population with the CNV genotypes and allele frequencies found by PCR, and with the genotype frequency of tag SNP rs4112788.

*Isolated populations.

# Populations with values significantly low for a copy-number loss by our algorithm.

The Agilent H244K array contains six probes covering the 32 kb deletion involving the *LCE3C* and *LCE3B* genes, that has been associated with psoriasis and other autoimmune diseases [16,28,29,32,33]. To evaluate the reliability of the CNV analysis by aCGH in this region we expanded the analysis of *LCE3C_LCE3B-del* by using 8 additional probes, 4 on each side. Only three probes of the deleted region (involving *LCE3C)* consistently and reliably detected the *LCE3C_LCE3B-del* (Additional file 2: Figure S3). Although the variable probe intensities might suggest population-specific breakpoints for this CNV, it is most plausible that these differences reflect the variation in the hybridization efficiency of the probes in the array.

We individually genotyped the *LCE3C_LCE3B-del* for all the samples available from the 13 populations used in the aCGH analysis. There was good concordance of the aCGH intensity data in pooled population samples with direct PCR analysis of the *LCE3C_LCE3B-del* in individual samples, showing a lower frequency of the deletion in the samples with weak hybridization signals with respect to the YRI population (Table 1). In particular, the correlation (Spearman rho coefficient) between the aCGH log_2_ ratio values and the frequency of deleted allele was 0.857 (p-value = 0.0002). The highest frequency of the deletion was detected in the PIMA population, with an allele deletion frequency of 75% (log_2_ ratio in the sample pool of −0.67). The ALG population also had a high frequency of the *LCE3C_LCE3B-del* (67% with an intensity log_2_ ratio of −0.30). As expected from the aCGH data, Sub-Saharan populations have low frequencies of the deleted allele (28% in Bantu (BAN), 34% in YRI, and 35% in Pygmies (PYG)). However, two Asian populations (Hazara (HAZ) and Yakut (YAK)) have a *LCE3C_LCE3B-del* frequency lower than that of Sub-Saharan Africans (21% in HAZ and 23% in YAK), although the small number of samples for these two population groups (17 and 11 samples) made the estimation of their low frequency unreliable. The frequency of the *LCE3C_LCE3B-del* in the other populations varied between 50% and 62%. We estimated the Hardy-Weinberg equilibrium for each of the 13 populations and no significant deviations were observed, suggesting that genotyping errors were scarce (Tables 1 and 2).

**Table 2:** ***LCE3C_LCE3B-del *and rs4112788G genotype frequencies in 31 world populations**

**Main Geographic Region**	**Populations (ID)**	**Individuals**	**HWE**		**(%) allele frequencies LCE3 C_LCE3B-del**		**SNP genotype frequencies (%)**			**LD**
			**x**^**2**^	**p-value**	**+**	**−**	**AA**	**AG**	**GG**	**r**^**2**^
Sub-Saharan Africans	Pygmies* (PYG)	44	0.93	0.33	64.77	35.23	0	13.64	86.36	0.013
	Mandenka (MAN)	17	0.78	0.56	58.82	41.18	4.55	22.73	72.73	0.121
	Yoruba (YRI)	22	2.18	0.14	65.91	34.09	0	28.57	71.43	0.089
	Bantu (BAN)	18	2.66	0.10	72.22	27.78	0	50.00	50.00	0
North-African/Middle East	Mozabite* (ALG)	26	0.04	0.84	32.69	67.31	0	21.43	78.57	0.256
	Bedouin* (BED)	36	0.47	0.49	47.22	52.78	14.63	53.66	31.71	0.772
	Druze* (DRU)	37	1.87	0.17	36.49	63.51	9.09	56.82	34.09	1
	Palestinian (PAL)	40	0.44	0.50	31.25	68.75	4.44	44.44	51.11	0.936
European	French (FRA)	27	1.47	0.23	40.74	59.26	16.67	41.67	41.67	1
	French Basque* (BASQ)	21	1.70	0.19	40.48	59.52	9.09	54.55	36.36	0.91
	Sardinian* (SARD)	24	0	0.97	29.17	70.83	13.04	47.83	39.13	1
	Italian (ITL)	17	0.28	0.60	31.76	38.24	35.00	40.00	25.00	1
	Orcadian* (ORC)	16	2.80	0.09	31.25	68.75	23.08	23.08	53.85	1
	Russian (RUS)	23	0.18	0.68	39.13	60.87	14.29	42.86	42.86	0.897
Central-South Asians	Brahui (BRA)	22	0.61	0.44	43.18	56.82	21.74	43.48	34.78	1
	Balochi (BAL)	18	0.21	0.65	27.78	72.22	5.00	40.00	55.00	0.852
	Makrani (MAK)	24	0.67	0.41	50.00	50.00	23.81	42.86	33.33	0.55
	Sindhi (SIN)	21	1.71	0.19	33.33	66.67	0	50.00	50.00	0.905
	Kalash* (KAL)	24	1.53	0.22	47.92	52.08	16.67	55.56	27.78	0.861
	Burusho (BUR)	23	3.19	0.07	41.30	58.70	27.27	31.82	40.91	1
	Hazara ( HAZ)	17	1.14	0.29	79.41	20.59	43.75	56.25	0	0.904
Eastern Asians	Han (CHB)	34	0	0.98	38.24	61.76	0	52.17	47.83	0.468
	Japanese (JPN)	26	2.45	0.12	48.08	51.92	50.00	37.93	37.93	0.837
	Yaut* (YAK)	11	0.55	0.46	77.27	22.73	50.00	31.82	18.18	1
	NEA	34	0.54	0.46	45.59	54.41	20.59	52.94	26.47	0.852
	SEA	36	0.73	0.40	29.17	70.83	5.88	50.00	44.12	0.537
Oceania	Papuan-Melasian (OCE)	35	2.31	0.13	50.00	50.00	26.47	41.18	32.35	0.935
Americans	PIMA	22	0.51	0.48	25.00	75.00	9.09	36.36	54.55	0.804
	MAYA	21	0.94	0.33	38.10	61.90	7.14	57.14	35.71	1
	Karitiana (KAR)	17	0.02	0.90	97.06	2.94	90.48	4.76	4.76	1
	Surui* (SUR)	15	1.52	0.22	26.67	73.33	10.53	26.31	63.16	1

*Isolated populations.

Allele and SNP genotype frequencies for all populations analysed, and the *r*^*2*^ value obtained in the linkage disequilibrium analysis.

**Figure 1:**
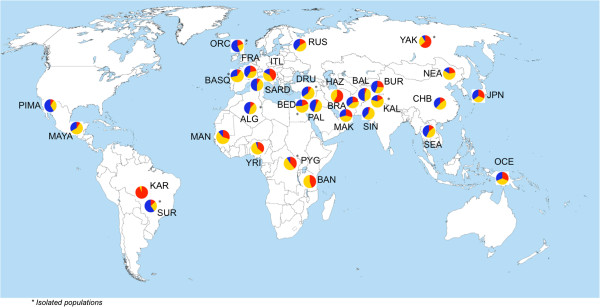
**Global distribution of the genotype frequencies for the *LCE3C_LCE3B *CNV.** The *LCE3C_LCE3B-del* is found in all the populations studied, mostly in the heterozygous state (yellow). The frequency of *LCE3C_LCE3B-del* in the homozygous state (blue) is higher in most non-Sub-Saharan African populations, with the exception of few ethnic groups. By contrast, the non-deletion homozygous state (red) tends to be more frequent in Sub-Saharan Africans, with the exception of few ethnic groups (KAR, ITL and YAK).

**Figure 2:**
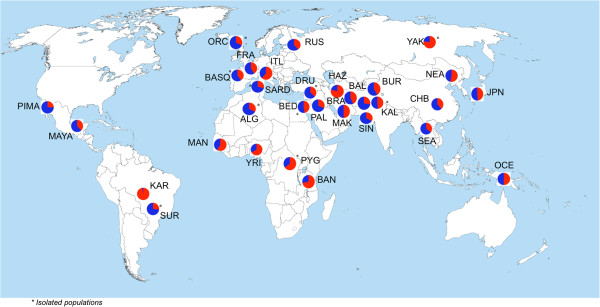
**Global distribution of allele frequencies for *LCE3C_LCE3B *CNV.** The deleted allele (blue) is more frequent in non-Sub-Saharan African populations than in Sub-Saharan Africans, with the exception of some ethnic groups (KAR, ITL and YAK).

We then evaluated the frequency of *LCE3C_LCE3B-del* in almost all available samples from the HGDP, representing a large worldwide diversity (768 genotyped samples from 31 populations). *LCE3C_LCE3B-del* was found to be common in most populations even though in a heterozygous state (Figure 1). By contrast, remarkable differences between groups were observed in the homozygote frequency of the deleted and non-deleted alleles. Apart from a few populations, Sub-Saharan Africans tend to have a lower frequency of the deleted allele in the homozygous state, which was significantly higher in the rest of the populations (Figure 2). Hence, excluding the few exceptional populations, these results generally suggest a possible non-Sub-Saharan African sweep for this locus. Thus, it is possible that the homozygous deletion has swept almost to fixation in mostly non-Sub-Saharan African populations, probably as a consequence of genetic drift produced by a bottleneck during the human expansion “Out-of-Africa”. The lowest frequency of *LCE3C_LCE3B-del* (3%) was found in the Karitiana (KAR) population from the Amazon region of Brazil.

The small sample sets of some of the populations studied here may be responsible for dramatic differences observed in the frequencies of the *LCE3C_LCE3B-del* between populations, such as the Surui (SUR), KAR and YAK. However, these ethnic groups are geographically isolated and they are less likely to have suffered genetic mixture from other populations. It is known that specific demographic histories may be key factors to explain allele frequencies among human ethnic groups [51,52] and therefore, small geographic clines could be caused by a long-standing history of spatially restricted gene flow. This would imply that although there are continental-scale clusters or general selective sweeps, such as the non-Sub-Saharan African sweep, allele frequencies change gradually on small geographic scales. Thus, the populations that we found to have exceptional frequencies of the deleted or non-deleted allele could represent a clear example of a particular genetic drift or relatively recent selection event that generated extremely large differences.

### Linkage disequilibrium of *LCE3C_LCE3B-del* with a neighbouring SNP

We evaluated the linkage disequilibrium (LD) between the tag SNP rs4112788 and the *LCE3C*_*LCE3B* 32-kb deletion [16] in all the populations analysed. We found that rs4112788*A* is associated with the non-deleted CNV allele in all cases, while rs4112788*G* can be associated with both CNV non-deleted and deleted alleles. While the first case is valid for all the populations analysed, the latter association differed between groups.

There is a high frequency of homozygosity for rs4112788*G* in sub-Saharan African individuals but not for the *LCE3C_LCE3B-del* (Figure 2, Table 1). This results in the lowest LD between rs4112788*G* and *LCE3C_LCE3B-del*, ranging between 0.01 and 0.12. The high frequency of rs4112788*G* in the ALG population was correlated with a higher frequency of the *LCE3C_LCE3B-del*, even though the *r*^*2*^ value remains low (0.26). We found a high concordance between the frequency of rs4112788*G* and the *LCE3C_LCE3B-del* in most of the other populations, with *r*^*2*^ values >0.8 in most of them. Some exceptions with *r*^*2*^ values around 0.5 were the CHB (0.47) and South East Asians (0.54), YAK, Makrani (MAK) and HAZ populations (Table 2).

## Discussion

In this study, we first documented the distribution of regions with variable copy number between twelve populations, comparing them with a Sub-Saharan African population (YRI) by aCGH, and using two independent CNV algorithms. We used stringent criteria to define a CNV based on the log_2_ ratios above 0.25 (both direct and dye-swap labels) for 3 consecutive probes in at least one of the comparison experiments, and concordance with the two CNV algorithms. We focussed on CNV of at least 30-kb in order to identify relative large polymorphisms that could present a specific pattern of variation among human populations.

Previous studies of samples in the HapMap collection and the HGDP panel highlighted a gradual decrease in genetic diversity in function of the distance from Sub-Saharan Africa [24,36-40], a result that reflects the influence of geography on human genetics and that is consistent with the serial-founder model of human expansion out of Sub-Saharan Africa. These African populations have the highest degree of heterozygosity in most genomic regions, while populations from America and Oceania present the lowest intra-population variance. By using a pooled approach we looked to dilute intra-population differences and to enhance inter-population variability. Hence, comparing different pools of individuals from different populations with a pool from an African population, we expected to find more CNV as the geographic distance from Africa increases. Indeed, we found 54 loci that show structural variation in the form of CNV in worldwide populations (Additional file 2: Figure S1), most specifically in populations from America and Oceania, followed by Eastern Asian and Western Asian populations (Additional file 2: Figure S2).

Interestingly, we found an enrichment of segmental duplications (SD) in the loci detected and 58 known genes totally or partially overlapping some CNV regions (Additional file 3: Table S3). Most of these genes are involved in sensory perception, the immune system and distinct metabolic pathways, and some are associated with disease. These results are consistent with previous reports [1,3], and they clearly support the idea that population-specific CNV profiles could explain adaptations to environmental pressure and differences in disease prevalence among populations.

Due to the significant association of *LCE3C_LCE3B-del* to the susceptibility to some autoimmune diseases that have a higher prevalence in some populations from developed countries, such as psoriasis, we expected to find differences in the frequency of the deletion among populations with different geographic origins and demographic histories. Re-analysing the aCGH data for the *LCE3C_LCE3B* region, we observed differences in signal intensity in a region smaller than the 6 probes that cover the 32-kb deletion. This could suggest population differences in the CNV breakpoints, particularly since the Database of Genomic Variants currently lists more than 25 distinct large structural variants that span either the *LCE3C* and *LCE3B* genes, or both, and the specific breakpoint coordinates used in our study correspond to one deletion identified for the first time in a European population. However, the same breakpoints were also used successfully in some Asian groups. Furthermore, it is important to consider that some of the probes in the array might not be absolutely specific and they may hybridise to similar sequences, probably other LCE genes, masking the signal from similar regions. Indeed, it is known that ascertaining CNV by aCGH is complicated due to poor power and non-trivial rates of false positives. Moreover, using genome-wide scanning techniques to detect CNV, like the Agilent H244k aCGH array, have a limited capacity to characterize specific breakpoints. In a population survey of the frequency of the deletion by PCR, we could amplify the deleted and non-deleted allele in all populations and samples, which confirms the limited power of the aCGH platform to characterize specific breakpoints. Although other unmeasured CNVs may affect this region in some populations, our analysis indicates that the specific deletion studied here may be the predominant one in most populations.

We found most populations to have a high percentage of the deleted allele, mostly in the heterozygous state, with the exception of some isolated instances and Karitiana population, which present a high number of relatives pairs that could reduce the representativeness of allele and genotype frequencies in this population [53]. The aCGH results indicate that most populations tend to have a higher frequency of the deleted allele than Sub-Saharan Africans. The high frequency of the deletion could reflect some selection for the deletion among human populations, even though it has recently been described as a susceptibility factor for psoriasis and other autoimmune diseases [16,29,32-34]. Thus, the 32-kb *LCE3C_LCE3B*-*del* could offer protection against an unknown element, and its role as a susceptibility factor for autoimmune inflammatory diseases may be a “new” consequence of this earlier adaptation. In other words, the LCE cluster could had been subjected to natural selection at different times during human evolution, and a partial sweep of the deletion could occur if individuals carrying the deleted allele had greater resistance to specific pathogens (for example). In such scenario, however, the fitness advantage would have to outweigh the loss of the *LCE3C_LCE3B* genes and the potential regulatory changes of the LCE cluster incurred by disruption to the surrounding genomic region. A similar scenario has been put forward for rearrangements associated with the alpha-globin gene family, where recurrent deletions of *HBA1* and *HBA2* associated with alpha-thalassemia have reached a high frequency in Mediterranean and Pacific populations [54]. Moreover, it is important to take into account that the autoimmune diseases related to *LCE3C_LCE3B-del* are also thought to be associated to other genetic variants [55-57], and that an important environmental component is involved in these disorders [58-61]. Thus, *LCE3C_LCE3B-del* only represents another genetic factor involved in susceptibility to these diseases, together with environmental factors like infections, drugs, stress, smoking and climate.

Populations in the HGDP have different sample sizes and, in some of them, first and/or second degree relatives pairs have been detected [53], which could both influence the estimation of the true values of allele and genotypes frequencies that underlie several studied populations. Furthermore, they present different demographic histories, and all these factors may also affect the power to detect selection. We should take into account the possibility that genetic differences among human populations could be caused by neutral demographic processes, such as “allele surfing”. This phenomenon is the result of the intense amount of genetic drift produced by strong bottlenecks that occurred during the exit “out of Africa”, which was followed by a spatial expansion that could lead to the geographic spread of an allele and increase its frequency in newly colonised areas [62]. This neutral process has recently received special attention due to its consequences for allele frequencies that appear to reflect a selective process. Thus, a definitive evidence of the influence of the natural selection on genetic population differences it is not currently available. For example, two reports described an increase in the frequency of a derived allele outside Africa for two genes involved in the control of brain size (*MCPH1* and *ASPM)*, and high LDs [63,64]. It was proposed that the derived haplotypes might be under local positive selection in non-African populations, although it was recently demonstrated that neutral allele surfing could generate similar geographic distributions of allele frequencies during the range expansion of Africa [65].

It is clear that the deleted allele has been established in most world populations, which probably has some kind of functional consequences. Expression of the *LCE3C* and *LCE3B* genes is induced upon epidermal activation as a consequence of inflammation or skin disease [16]. However, the high frequency of the deletion worldwide suggests the existence of some redundancy in the function of *LCE* genes in this cluster. It is possible that other genes fulfil the function of *LCE3C* and *LCE3B,* although imperfectly, contributing to the abnormal differentiation and epidermal hyperproliferation characteristic of psoriatic lesions. Thus, when other susceptibility components are not present, the deletion is insufficient to produce the abnormal phenotype but when several susceptibility components concur, the *LCE3C_LCE3B-del* could lead to disease development.

In the previous study identifying the 32-kb deletion associated with psoriasis in several populations of European ancestry, 14 SNPs were found related with *LCE3C_LCE3B-del*, with allele *G* at rs4112788 being the only one in strong LD [16]. Despite we could contemplate, *a priori,* the possibility that the association between these SNPs and the *LCE3C_LCE3B-del* could vary among populations, a strong LD between a given SNP and CNV suggest a single origin of both variants. For this reason we did not expect to find strong association between other SNPs and the *LCE3C_LCE3B-del* in other populations.

A strong LD might be also a common feature of a biallelic CNV, which is particularly useful in association studies for complex disorders in which the redundancy of information implied by LD can be used to optimize genotyping. Nevertheless, this might not be useful in association studies of all populations, since LD patterns vary among populations of different geographic origin and a much higher proportion of *r*^*2*^ variance could be attributed to differences between continental regions [66], with similar characteristics found in CNV. Specifically, increases in LD as the geographic distance from East Sub-Saharan Africa augments have been reported, with the highest values occurring in the Americas, followed by Oceania, East Asia, Eurasia and Africa. As for CNV, this pattern matches the prediction from a model of sequential founder effects during spatial expansion from Africa, given that such founder effects would be expected to increase the LD at each step of the expansion [24,67].

Our results for *LCE3C_LCE3B-del* are consistent with previous studies showing that the extent of LD in non-Africans is higher than in Africans [68], reflecting the origin and spread of modern humans from Africa. We found *r*^*2*^ values >0.8 in all non-African populations with the exception of two Chinese groups (CHB and population grouped as SEA) and the Makrani population. Although these exceptions are not defined as “isolated” in the HGDP, they may reflect a particular demographic and genetic history of these populations or alternatively, a bias due to the small number of individuals from these populations in the study. However, the LD pattern between rs4112788*G* and *LCE3C_LCE3B-del* found for all populations differs from other studies. While the trend observed for general LD consists of a successive increase in the LD in Middle East-North Africa, Central South Asia, Europe, East Asia, Oceania and America with respect to Sub-Saharan Africa, we essentially detect low *r*^*2*^ values in African populations and similar high values for the rest of the world.

## Conclusions

Our results show a rise in the number of CNV as the geographic distance from Africa increases, reflecting the influence of geography on human genetics. The higher frequency of *LCE3C_LCE3B-del* found in most of non-Sub-Saharan African populations suggests the fixation of the deleted allele as a consequence of the genetic drift that occurred during the exit from Africa. The CNV deleted allele has been associated with susceptibility to autoimmune diseases, not only implying that natural selection but that neutral demographic processes can define the differences in disease frequencies and phenotypic diversity among human populations.

Population genetics has the power to provide insights into the demographic history of populations, the selective pressures acting on genetic variation and the mutational processes generating diversity. With NGS able to precisely define the many types and forms of CNV, and other structural variations, we would expect a rapid advance in the discovery and characterisation of novel variants over the next few years. The analysis in patient samples and in subjects of different populations will define their evolutionary history and/or their role in human adaptation and disease.

## Methods

### Array comparative genomic hybridisation (aCGH)

To investigate the variability of CNV in human populations we used the Human Genome CGH Microarray Kit 244 k (Agilent Technologies Inc, Santa Clara, CA, US), which contains over 244,000 probes and covers the entire genome at 10 kb resolution. The initial detection of CNV was performed using a DNA-pooling approach. Experiments were performed in duplicate with DNA labelling colour reversal (dye-swap). The specific experimental protocol that we used was based on the Agilent Oligonucleotide Array-Based CGH for Genomic DNA Analysis*.*

We selected 86 DNA samples from two populations of the HapMap Collection and 343 DNA samples from 21 ethnic groups of the Human Genome Diversity Panel-Centre d’Etude du Polymorphisme Humain (HGDP-CEPH) grouped into 11 populations. Each population group contained samples from 20 to 50 individuals (Additional file 4: Table S1 and Additional file 1). We generated 13 DNA pools (one for each population), which included all the samples available for each population group (the data analysis procedure is explained in the Additional file 1). The final DNA concentration of the pooled samples was measured on a NanoDrop® ND-1000 UV–vis Spectrophotometer (Wilmington, Delaware, USA), and DNA integrity was evaluated by electrophoresis in alkaline gels*.*

### Analysis of gene content in regions found by aCGH

CNV gene content was determined with the RefSeq gene annotation from the UCSC Genome Browser. To measure the enrichment of genes located within CNV that show population differences in comparison with the rest of genes in the human genome, we analysed the enrichment of GO categories using the Gene Set Analysis Toolkit V2 (http://bioinfo.vanderbilt.edu/webgestalt/).

### Multiplex PCR-based genotyping

To analyse the specific *LCE3C_LCE3B-del* region by PCR, we used the same samples as those used in the aCGH, with the exception of the two HapMap populations (YRI and CHB) that were replaced by samples of the same ethnic group but from HGDP-CEPH (Yoruba from Nigeria and Han Chinese). In addition, we included 25 new populations for this analysis, also from HGDP-CEPH, grouped into 18 ethnic groups (Additional file 1). Each population group had samples from between 17 and 44 individuals. In combining samples we took into account the geographic location and proximity, grouping small numbers of individuals from different populations (mostly Chinese samples). Information about the groups, their identification (ID) and the number of samples are shown in the Additional file 1 and Additional file 5: Table S2. For the CNV genotyping assay, we used individual samples instead of pools in order to determine not only the inter-population differences but also, the intra-population variability.

The *LCE3C_LCE3B-del* region, encompassing 32,199 bp (chr1:150,822,166-150,854,365 in hg18) was genotyped by multiplex PCR with 5′FAM modification, subsequent capillary electrophoresis and Gene Mapper analysis (Gene Mapper v.4.0, Applied Biosystems), as described previously [16]. Briefly, the DNA template was amplified simultaneously with two forward and two reverse primers, and the PCR products were diluted, added to a formamide-ROX mixture and subsequently resolved by capillary electrophoresis (3730xl DNA Analyzer; Applied Biosystems). Correlation between CNV deletion and aCGH was explored using the Spearman Rho test (Stata v10 software).

### SNP genotyping assay

The rs4112788 SNP was genotyped using a C_31910050_10 TaqMan® Pre-Designed SNP Genotyping Assay (Applied Biosystems, Foster City, CA). PCR amplification was performed according to the product specifications and alleles were discriminated on a 7900HT Fast Real-Time PCR system, analysing the data with SDS 2.3 package (Applied Biosystems). When genotyping the SNP, a total of 56 samples from different populations were no longer available due to the poor quality of the DNA and therefore, they were not analysed (Additional file 5: Table S2). LD Measurements and haplotype association statistics were calculated using Haploview software.

## Competing interests

The authors declare that they have no competing interests.

## Authors’ contributions

LB designed the study, carried out all the experimental work and data analysis, and drafted the manuscript. ERM designed the PCR genotyping experiments and helped with the experimental work. MGA carried out the aCHG array experiments. JRG carried out the computational analysis of CNVs using GADA software. MC helped with the data analysis and biological interpretation of the results. LA developed the algorithm for CNV detection using aCGH data and helped with the data analysis. XE designed the study and revised the manuscript. All the authors read and approved the manuscript.

## Supplementary Material

Additional file 1 Supplementary Methods. Click here for file

Additional file 2 Supplementary Figures. Click here for file

Additional file 3 **Supplementary Table 3.** Log_2_ ratio values for each CNV locus (coordinates in hg18) in the populations analysed by aCGH against the Yoruba population. Information about the genes and SD overlapping these loci. Click here for file

Additional file 4 **Supplementary Table 1.** Population used by aCGH analysis. Click here for file

Additional file 5 **Supplementary Table 2.** Population used in LCE3C_LCE3B-del genotyping. Click here for file
