# Supplementary material for: Worldwide population distribution of the common LCE3C-LCE3B deletion associated with psoriasis and other autoimmune disorders
Source: BMC Genomics. 2013 Apr 17;14:261. doi: 10.1186/1471-2164-14-261 (PMC3639927; doi:10.1186/1471-2164-14-261)
Supplement: Additional file 1 — Supplementary Methods. [file 1471-2164-14-261-S1.pdf]

SUPPLEMENTARY METHODS

Array-based Comparative Genomic Hybridisation (aCGH)

Samples Selection. We carried out an array-based Comparative Genomic Hybridization (aCGH) analysis on pooled samples from different populations of the HapMap Collection and Human Genome Diversity Panel-Centre d'Etude Du Polymorphisme Humain (HGDP-CEPH). To obtain the pooled samples of HapMap we used DNA extracted from lymphoblastoid cell lines (LCLs) obtained from the Coriell Institute for Medical Research, and for the HGDP-CEPH we used DNA extracted from LCLs obtained from the Foundation Jean Dausset-CEPH. The HapMap samples consisted of 86 individuals from two different populations: 40 Yoruba individuals from Ibadan, Nigeria (YRI), and 46 Han Chinese from Beijing, China (CHB). Samples from HGDP-CEPH consisted of 20 Bantu from Kenya and South Africa (grouped together as BAN), 51 Pygmy from Central African Republic and Democratic Republic of Congo (grouped together as PYG), 30 Mozabite from Algeria (ALG), 29 French from France (FRA), 49 Bedouin from Israel (Negev: BED), 25 Brahui (BRA) and 25 Hazara (HAZ) from Pakistan, 25 Yakut (YAK) from Siberia, 39 Papua and Melanesian (grouped together as OCE) from New Guinea and Bougainville, and finally 25 Pima (PIMA) and 25 Maya (MAYA) from Mexico (Supplementary Table 1).

The 21 ethnic groups selected were re-grouped into 13 populations based on geographic proximity. In the case of Bantu individuals, who are from different countries and are described as different sub-ethnic groups, we grouped all of them as general Bantu speakers because of the small size of each sub-group. For general analysis we further grouped the populations into seven main geographic regions (Supplementary Table 1). In addition, we took into account that 5 of these ethnic groups (corresponding to 4 populations) were considered as isolated groups with distinct cultural, linguistic, demographic or genetic features [1, 2].

CGH data analysis. Before analysing the data, we used a dye-normalized protocol to balance the fluorescent intensities of the two dyes (green Cy3 and red Cy5 dye) and to compare the results from different experiments. We used a Global Lowess strategy (*BACANAL* package: Lozano et al. unpublished) to correct the spatial experimental effects and thus, to obtain good comparable intensities for each hybridisation. Each experiment consisted in comparing each of the 12 population groups against the Yoruba population (Supplementary Table 1) used as a reference.

Copy number changes were detected using two different algorithms. First, we used a Perl script to identify regions where a stretch of consecutives probes had values indicative of copy number changes. These genomic imbalances were determined based on the average $\log_2$ of the Cy5/Cy3 ratios of the spotted replicates, and the regions were considered as amplified or deleted when probes exceeded +/-0.3-fold the threshold. In other words, an "entry probe" was first selected on the basis of its $\log_2$ ratio, if equal or greater than 0.3 (green squares) or equal or less than -0.3 (red squares), and of opposite sign in direct and dye swap (reverse) assays. We discarded probes that exhibited discordant $\log_2$ ratios between both dyes. The CNV loop was extended if consecutive probes showed $\log_{2r}$ ratios compatible with a copy number gain –or amplification- (≥0.25) or loss –deletion- (≤-0.25), in the same direction as the entry probe, and in the direct and dye swap experiments. The CNV was considered as extinguished when none of the probes in the direct and reverse experiments was above or below the extension threshold. We only retained CNVs that were associated with at least three altered probes (including entry probe) in each experiment that compared populations.

Second, we used a GADA algorithm [3, 4] that relies upon a piecewise-constant vector representation of the intensity data in order to facilitate extremely fast matrix-based breakpoint finding in two primary steps. The first step is a Bayesian learning process that generates a list of candidate breakpoints and segment means, while trying to strike an optimal balance between model fit and model sparseness (the number of breakpoints). After an initial segmentation

process, a "t" statistic is calculated for each segment as a function of the segment mean and variance. The second step is then a backwards elimination process which removes breakpoints with a level of significance (t statistic) less than the user-defined threshold, T. In our configuration we used T=4.5 and MinSegLen=3 (minimum number of consecutive probes to determine an alteration).

Multiplex PCR-based genotyping

Samples selection. We carried out Multiplex PCR-based genotyping to analyze specific *LCE3C_LCE3B-del* region in all the populations. For this analysis the samples came from Human Genome Diversity Panel cell lines, using DNA extracted from LCLs obtained from the Foundation Jean Dausset-CEPH.

Samples consisted of 22 Yoruban from Nigeria (YRI), 44 Pygmy from Central African Republic and Democratic Republic of Congo (grouped together as PYG), 17 Mandenka from Senegal (MAN), 18 Bantu from Kenya and South Africa (grouped together as BAN), 26 Mozabite from Algeria (ALG), 36 Bedouin from Israel (Negev: BED), 37 Druze and 40 Palestinian from Israel (Carmel: DRU and PAL respectively); 22 Brahui (BRA), 18 Balochi (BAL), 17 Hazara (HAZ), 24 unrelated Makrani (MAK), 21 Sindhi (SIN), 24 Kalash (KAL) and 23 Burusho (BUR), all from Pakistan; 34 Han Chinese (CHB); 34 individuals from small Chinese ethnic groups (Mongola, Tu, Xibo and Hezhen) grouped as North-Eastern Asian (NEA); 36 from other small Chinese ethnic groups (Dai, Lahu, She, and Naxi) grouped as South-Eastern Asian (SEA); 11 Yakut (YAK) from Siberia; 26 Japanese (JPN); 35 Papua and Melanesian (grouped together as OCE) from New Guinea and Bougainville; 29 French (FRA) and 21 French Basque (BASQ); 24 Sardinian from Sardegna (SARD); 17 Tuscan and North-Italian individuals (from Bergamo) grouped together as ITL; 16 Orcadian from Orkney Islands (ORC); 23 Russian (RUS); 22 PIMA and 21 MAYA from Mexico; and finally, 17 Karitiana (KAR) and 15 Surui (SUR) from Brazil

(Supplementary Table 2). Differences in the number of individuals in the same populations used in the aCGH analysis are due to the absence of better quantity of DNA or its poor quality.

Several samples (96/1064) from HGDP-CEPH panel have been classified as “relatives”. It means that there are some populations containing pairs of samples in which a first and/or second degree of familiar relationship have been detected. The depth analysis of relative pairs existing in each population has provided by Rosenberg [5]. Populations with higher number of relatives are Pygmies, Melanesian, and Central-South American ethnic groups (Rosenberg [5] Supplementary Material).

References

1. Rosenberg NA, Pritchard JK, Weber JL, Cann HM, Kidd KK, Zhivotovsky LA, Feldman MW: **Genetic structure of human populations**. *Science* 2002, **298**(5602):2381-2385.
2. Cann HM, de Toma C, Cazes L, Legrand MF, Morel V, Piouffre L, Bodmer J, Bodmer WF, Bonne-Tamir B, Cambon-Thomsen A *et al*: **A human genome diversity cell line panel**. *Science* 2002, **296**(5566):261-262.
3. Pique-Regi R, Monso-Varona J, Ortega A, Seeger RC, Triche TJ, Asgharzadeh S: **Sparse representation and Bayesian detection of genome copy number alterations from microarray data**. *Bioinformatics* 2008, **24**(3):309-318.
4. Pique-Regi R, Caceres A, Gonzalez JR: **R-Gada: a fast and flexible pipeline for copy number analysis in association studies**. *BMC bioinformatics* 2010, **11**:380.
