# Supplementary material for: Worldwide population distribution of the common LCE3C-LCE3B deletion associated with psoriasis and other autoimmune disorders
Source: BMC Genomics. 2013 Apr 17;14:261. doi: 10.1186/1471-2164-14-261 (PMC3639927; doi:10.1186/1471-2164-14-261)
Supplement: Additional file 2 — Supplementary Figures. [file 1471-2164-14-261-S2.pdf]

SUPPLEMENTARY FIGURES

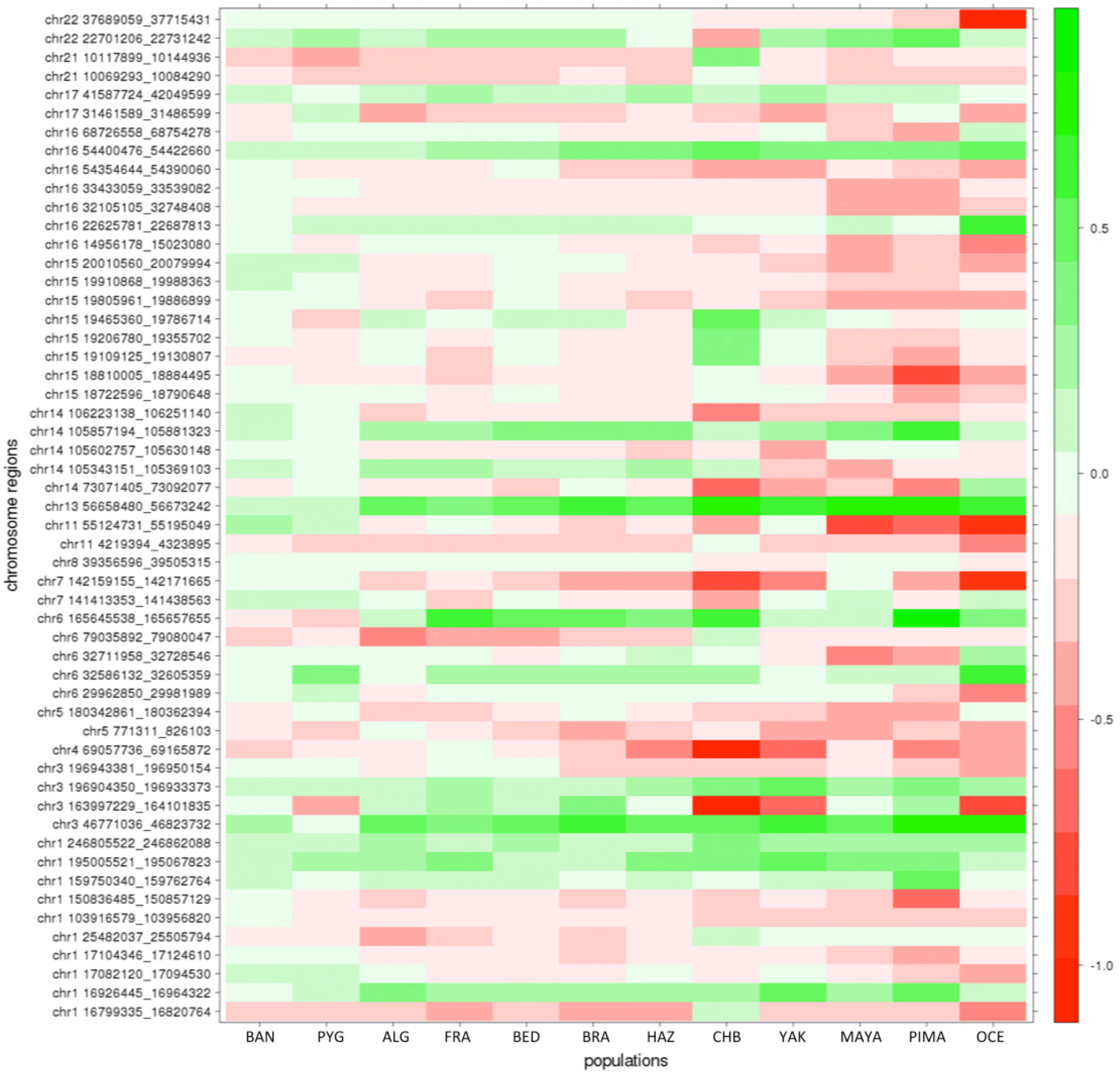

Supplementary Figure 1. Representation of the population specific CNVs according to the $\log_2$ ratio intensity values (aCGH)

Representation of the 54 regions found by aCGH analysis whose signal intensities (i.e. copy number) showed a distinct distribution in at least one of the 12 populations when compared to the Yoruba population. There is a gradual increase in signal intensity in function of the distance from the sub-Saharan Africa Y-axis: CNV coordinates in hg18; x-axis: populations by ID name.

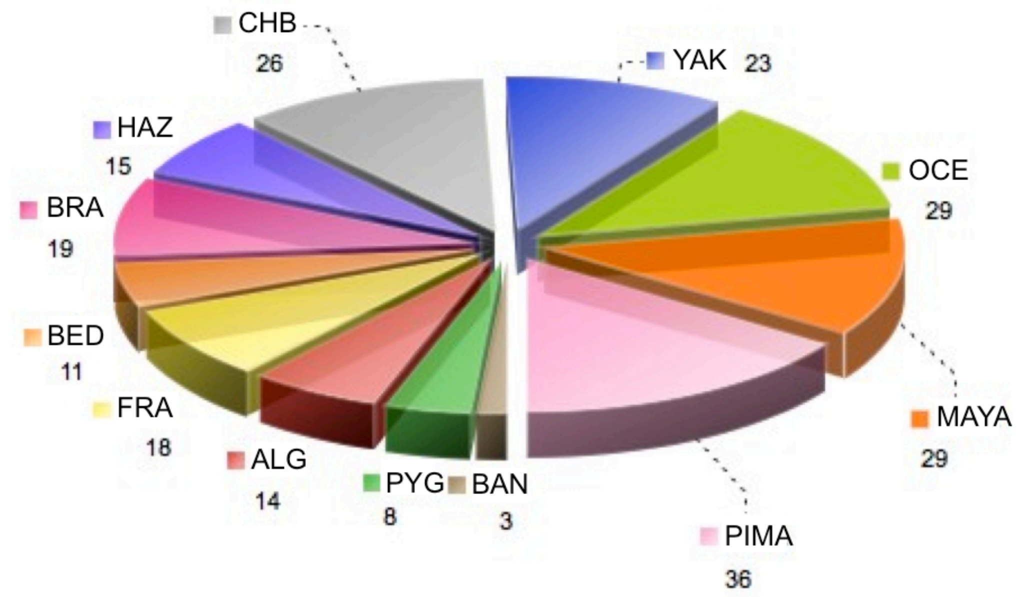

Supplementary Figure 2. Distribution of the number of CNVs found in each population in comparison to the Yoruba population.

Populations from America, Oceania and Eastern-Asia have more regions with copy-number differences with respect to Sub-Saharan Africans, Europeans, Northern African and Middle East populations.

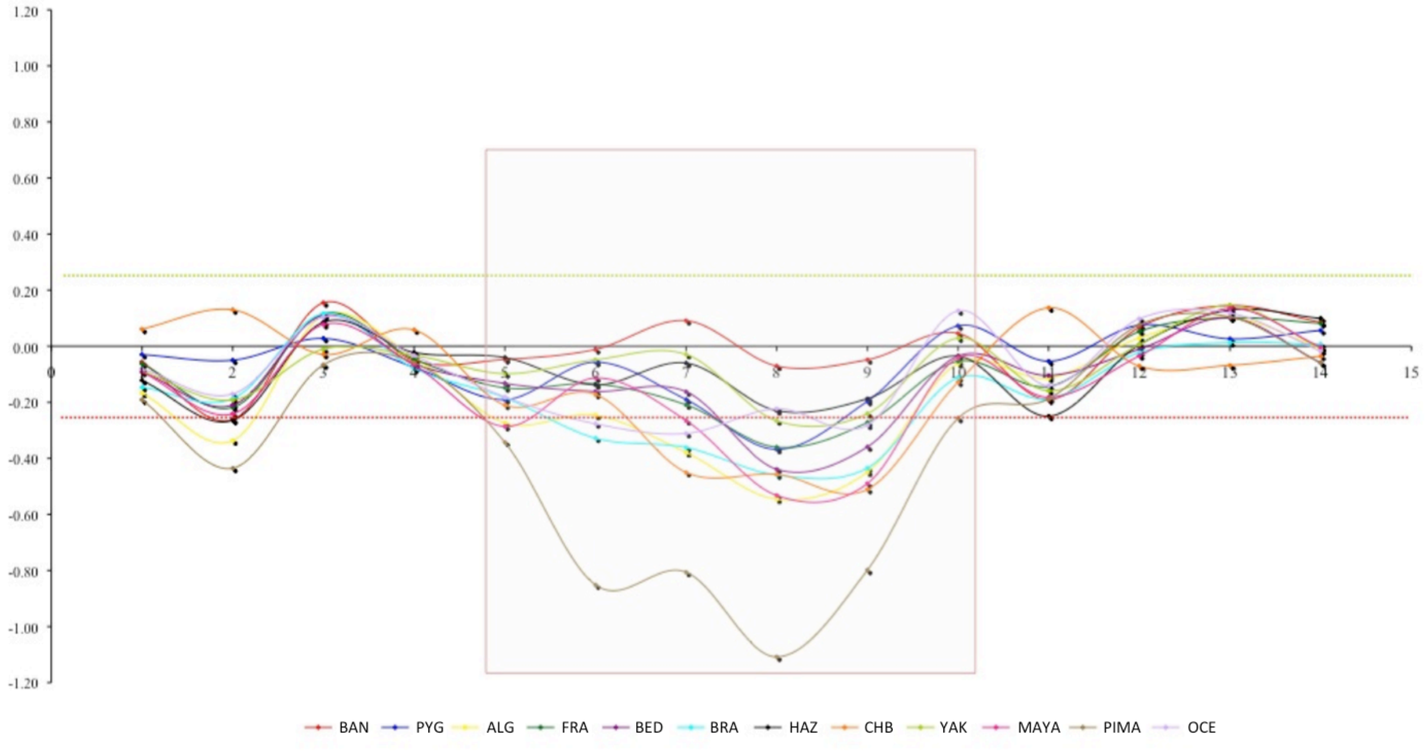

Supplementary Figure 3. The aCGH log_2 ratio values for *LCE3C_LCE3B-del*.

The log_2 ratios of the aCGH probes falling within the deletion are mostly negative when compared with the YRI population used as a reference. Y-axis: log_2 ratio values; x-axis: populations analyzed in comparison to YRI. Square: 6 probes falling within the deletion.
